# Plasmapheresis as an Alternative Treatment of Hypertriglyceridemia-Induced Pancreatitis: A Case Report

**DOI:** 10.7759/cureus.32000

**Published:** 2022-11-29

**Authors:** Afonso Santos, Filipa Ferreira, Catarina Brás, Andreia Curto, Mariana Silveira Ramos, Célia Madeira

**Affiliations:** 1 Nephrology, Hospital Professor Doutor Fernando Fonseca, Amadora, PRT; 2 Gastroenterology, Hospital Professor Doutor Fernando Fonseca, Amadora, PRT

**Keywords:** non-fractioned heparin, insuline, therapeutic plasma exchange (tpe), pancreatitis, hypertriglyceridemia, plasmapheresis

## Abstract

Hypertriglyceridemia-induced pancreatitis is a relatively common form of acute pancreatitis that may represent up to 10% of all etiologies of this condition. Due to its specific pathogenic mechanisms related to high serum triglyceride levels, different treatment options have been proposed, including insulin perfusion, heparin perfusion, and plasmapheresis. Although the superiority of plasmapheresis in this clinical setting has not been demonstrated in randomized clinical trials, many centers have reported its effectiveness and considered this as a possible alternative according to the current guidelines. We report a case of a young patient diagnosed with hypertriglyceridemia-induced pancreatitis that was successfully treated with plasmapheresis. Since complications associated with plasmapheresis are rare and other therapeutic options may not be so effective or safe, we believe that this should be a valid alternative treatment that may be offered to these patients. More studies are still needed to further evaluate its effectiveness and to elucidate if there is a subset of patients in whom treatment with plasmapheresis may be more beneficial.

## Introduction

Acute pancreatitis (AP) has multiple possible etiologies and hypertriglyceridemia (HTG) accounts for up to 10% of all cases [1,2]. In 15-20% of cases of severe HTG, usually defined as triglyceride levels above 1000 mg/dL, progression to AP may occur [2].

The exact mechanisms by which HTG induces pancreatitis are not known and diverse theories have been purposed, including the role of genetic, metabolic, and environmental causes [2,3]. The most accepted hypothesis suggests that the excess of chylomicrons in circulation causes occlusion in pancreatic capillaries inducing hydrolysis of these triglyceride-rich lipoproteins by pancreatic lipase into free fatty acids that lead to toxicity to the pancreatic endothelium and acinar tissue [4]. The hyperviscosity theory hypothesizes that accumulation of chylomicrons in the microcirculation reduces pancreatic capillary blood flow leading to ischemia [5]. Finally, the recognition of genetic polymorphisms as potential risk factors for the development of AP raised the hypothesis of genetic involvement in the pathogenesis of hypertriglyceridemia-induced pancreatitis (HTG-AP) [3]. Familial HTG may also lead to HTG-AP and autoantibodies against lipoprotein lipase (LPL) or its regulatory proteins may cause acquired chylomicronemia [6].

Apart from the supportive treatment that should be offered to all patients with AP, including intravenous hydration, analgesic medication, and a fastening state, it is important to consider other therapeutic measures directed to its specific etiology. In the setting of HTG-AP, different treatment options have been used, such as heparin, insulin infusion, and plasmapheresis [5]. Infusion of unfractionated heparin releases LPL attached to the endothelial cells, which leads to a transient reduction in serum triglycerides [7]. However, there are some risks associated with this therapy as it causes depletion of LPL on the surface of endothelial cells which may lead to a new increase in serum triglycerides [8]. Furthermore, it may increase the hemorrhagic risk in the setting of pancreatic necrosis [9]. Insulin infusion is other commonly used strategy as it promotes the synthesis of LPL from adipose and muscle cells; however, there is no clear evidence of its benefit in non-diabetic patients. The non-invasive nature of therapy with insulin is undoubtedly desirable but requires careful monitoring [2,5].

As mortality associated with HTG-AP may reach 30%, starting a more aggressive and rapidly efficient treatment such as plasmapheresis may be justified [6]. However, even in the setting of intensive care, HTG-AP is not a common indication for plasmapheresis [10-12]. According to the last published guidelines on therapeutic apheresis, HTG-AP constitutes an indication of category III (Grade 1C) for treatment with plasmapheresis, meaning that the optimum role of apheresis therapy is not established and decision-making should be individualized [6]. Extracorporeal elimination of large lipoproteins is hypothesized to stop further organ damage and it is thought that plasmapheresis can significantly decrease triglyceride levels, reduce inflammatory cytokines, and potentially replace deficient LPL when fresh frozen plasma (FFP) is used as replacement fluid. Reductions in triglyceride levels of 49-97% have been reported following a single procedure. Treatment goals rely on triglyceride reduction at least to mild-moderate levels, frequently defined as <500 mg/dL [6]. The main complications associated with plasmapheresis are hypotension, infection, hypersensibility reactions, bleeding, and hypocalcemia [11]. Herein, we present a case of HTG-AP successfully managed with plasmapheresis.

## Case presentation

A 38-year-old Asian male with a known history of hyperlipidemia presented in the emergency department with severe abdominal pain that started eight hours before admission. The patient denied any previous history of alcohol consumption or smoking and was not taking any medication. Vital signs at admission were as follows: blood pressure 135/85 mmHg, heart rate 117 beats per minute (bpm), respiratory rate 16 cycles per minute (cpm), and temperature 36.8ºC. Physical examination was positive for umbilical and right lumbar pain. Laboratory workup revealed normal serum amylase and severe HTG of 3170 mg/dL (Table 1).

**Table 1:** Laboratory evaluation at admission. CRP: c-reactive protein; HDL: high-density lipoprotein; LDL: low-density lipoprotein

Laboratory results	Value	Normal range
Hemoglobin (g/dL)	14.4	13-18
Leucocytes (cells/uL)	10,400	3,800-10,600
Neutrophils (cells/uL)	7,700	1,800-6,900
CRP (mg/dL)	2.29	<0.3
Lactate (mmol/L)	1.35	<1.80
Platelets (platelets/uL)	185	150-440
Amylase (U/L)	92.7	28-100
Total cholesterol (mg/dL)	567	<200
LDL cholesterol (mg/dL)	554	<115
HDL cholesterol (mg/dL)	13	>55
Triglycerides (mg/dL)	3,170	<150
Glucose (mg/dL)	179	60-100
Creatinine (mg/dL)	0.91	0.70-1.20
Urea (mg/dL)	23.2	<50.0
Calcium (mg/dL)	7.3	8.6-10.0
Ionized calcium (mmol/L)	1.2	1.15-1.35
Urine glucose	++++	0

An abdominal ultrasound was performed showing hepatic steatosis without other relevant findings. A computed tomography (CT) scan at admission showed globosity of pancreatic tissue and small volume of peri-pancreatic effusion. A diagnosis of HTG-AP was made and the patient was admitted to the gastroenterology ward. Upon the observation of severe HTG without hyperglycemia, it was decided to start treatment with plasmapheresis. A central venous catheter was placed and plasmapheresis treatment was started, using a PrismaFlex® monitor with a TPE 2000® filter (Lund, Sweden: Baxter International Inc.) (Table 2). Anticoagulation was prescribed with unfractionated heparin at a rate of 500 IU/hour.

**Table 2:** Plasmapheresis prescription details. Qb: blood flow rate; UFH: unfractionated heparin

Plasmapheresis prescription	
Filter	TPE 2000
Qb (mL/min)	200
Reposition fluid volume (mL)	3000
Reposition fluid rate (mL/h)	800
Reposition fluid	Albumin 5%
Treatment duration	3 h 45 min
Anticoagulation	UFH

**Figure 1:**
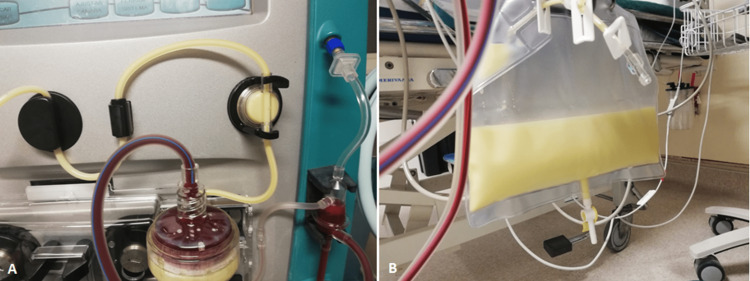
Filtered plasma (A) in the extracorporeal circuit and (B) in the effluent bag during plasmapheresis.

During plasmapheresis treatment, it was possible to document the milky appearance of the filtered plasma in the extracorporeal circuit and in the effluent bag (Figures 1A, 1B). There were no complications associated with the procedure. Approximately 12 h after treatment, the triglyceride levels were under 500 mg/dL, and symptoms progressively remitted. Imaging study by CT scan was repeated after 48 h revealing small areas of necrotic pancreatic tissue and increased volume of peri-pancreatic effusion. After 72 h, liquid diet (per os) was restarted and was well tolerated. During hospitalization, the patient kept blood glucose levels between 140-260 mg/dL and HbA1c was 9.5% leading to the diagnosis of diabetes mellitus.

The patient was discharged after one week of hospitalization and referred to endocrinology and gastroenterology consultations under treatment with bezafibrate and atorvastatin. Oral antidiabetic treatment was also started with metformin and sitagliptin. The evolution of triglycerides and total serum cholesterol, low-density lipoproteins (LDL), and high-density lipoproteins (HDL) levels after discharge are shown in Table 3.

**Table 3:** Lipid profile evaluation 12 h after plasmapheresis, at discharge, and three months after discharge. HDL: high-density lipoprotein; LDL: low-density lipoprotein; NA: not available

Lipid profile	12 h post-plasmapheresis	At discharge	After 3 months
Triglycerides (mg/dL)	457	521	690
Total cholesterol (mg/dL)	130	255	330
HDL cholesterol (mg/dL)	15	NA	38
LDL cholesterol (mg/dL)	115	NA	292

There were neither new hospitalizations nor the need for additional plasmapheresis sessions after six months of follow-up. The patient is currently treated with a combination of statin and fibrate, without adverse effects to report and with good lipid control.

## Discussion

HTG-AP may present as a life-threatening condition requiring aggressive treatment to control disease activity. Of note, in this case, amylase levels were always under the threshold of three times the upper limit of normal, as previously described in patients with HTG-AP [13,14]. Lipase measurement would be more specific than amylase for the diagnosis of pancreatitis, but it was not available in the emergency department laboratory panel. In this case, we emphasize the effectiveness of a single treatment with plasmapheresis in the reduction of HTG leading to a favorable outcome, with the first treatment reducing triglyceride levels by 85.6%. A milky appearance of the effluent is an expected finding, and it is a consequence of the high concentration of triglycerides in the filtered plasma. When available, we think plasmapheresis may be helpful in rapidly decreasing serum triglycerides and stopping the pathogenic pathways of HTG-AP. In this case, the patient had normal blood glucose levels at admission, which supported the decision of not starting insulin perfusion, since plasmapheresis was available as an alternative method. While lowering of triglyceride concentrations in the setting of HTG-AP is achievable with plasmapheresis, it is not known the exact extent of the benefit in terms of overall morbidity and mortality. Despite the marked reduction of HTG levels observed in this patient, the 48 h imaging studies revealed signs of worsening disease with small necrotic pancreatic areas. Although there are no randomized clinical trials showing its superiority compared to other treatments, other reports of the use of plasmapheresis in this clinical setting strongly suggest that it can be a safe and useful option for severe cases [7,15-19].

The Bi-TPAI trial, the first randomized clinical trial that compared insulin therapy to plasmapheresis showed no significant differences between both interventions. However, further studies are needed to elucidate the potential beneficial effects of plasmapheresis in the subgroup of patients with a suboptimal lowering of triglycerides with conservative treatment or patients with a severe course of pancreatitis [20,21].

Regarding long-term follow-up, the type of therapy used to treat pancreatitis seems to have no impact on the control of triglyceride levels. In this case, the patient had re-elevation of triglyceride levels three months after discharge. Knowing that statins and fibrates are two powerful medications against HTG, treatment with n-3 polyunsaturated fatty acids and nicotinic acid may also be considered [22]. In rare cases where triglyceride levels remain uncontrolled despite optimal therapy and pancreatitis recurs, plasmapheresis may be used as prevention treatment [23,24].

## Conclusions

HTG-AP may have a severe clinical course with mortality rates as high as 30% of cases. Different treatment options are available, such as insulin or heparin perfusion, but other therapeutic alternatives should be considered in the setting of severe cases. In this case, a single plasmapheresis session was effective and well-tolerated treatment, without associated complications.

Even if HTG-AP is a rare indication for this technique, plasmapheresis results in early and effective triglyceride reduction. However, large-scale studies are needed to further assess the efficacy of plasmapheresis and to help define subgroups of patients that may have higher benefits from this therapy.

## References

[1] Gavva C, Sarode R, Agrawal D, Burner J (2016). Therapeutic plasma exchange for hypertriglyceridemia induced pancreatitis: a rapid and practical approach. Transfus Apher Sci.

[2] Adiamah A, Psaltis E, Crook M, Lobo DN (2018). A systematic review of the epidemiology, pathophysiology and current management of hyperlipidaemic pancreatitis. Clin Nutr.

[3] Chang YT, Chang MC, Su TC (2008). Association of cystic fibrosis transmembrane conductance regulator (CFTR) mutation/variant/haplotype and tumor necrosis factor (TNF) promoter polymorphism in hyperlipidemic pancreatitis. Clin Chem.

[4] Havel RJ (1969). Pathogenesis, differentiation and management of hypertriglyceridemia. Adv Intern Med.

[5] Valdivielso P, Ramírez-Bueno A, Ewald N (2014). Current knowledge of hypertriglyceridemic pancreatitis. Eur J Intern Med.

[6] Padmanabhan A, Connelly-Smith L, Aqui N (2019). Guidelines on the use of therapeutic apheresis in clinical practice - evidence-based approach from the Writing Committee of the American Society for Apheresis: The Eighth Special Issue. J Clin Apher.

[7] Berger Z, Quera R, Poniachik J, Oksenberg D, Guerrero J (2001). Heparin and insulin treatment of acute pancreatitis caused by hypertriglyceridemia. Experience of 5 cases. [Article in Spanish]. Rev Med Chil.

[8] Watts GF, Cameron J, Henderson A, Richmond W (1991). Lipoprotein lipase deficiency due to long-term heparinization presenting as severe hypertriglyceridaemia in pregnancy. Postgrad Med J.

[9] Whayne TF Jr (2010). Concerns about heparin therapy for hypertriglyceridemia. Arch Intern Med.

[10] Lemaire A, Parquet N, Galicier L (2017). Plasma exchange in the intensive care unit: technical aspects and complications. J Clin Apher.

[11] Calça R, Gaspar A, Santos A, Aufico A, Freitas P, Coelho S (2020). Therapeutic plasma exchange in patients in a portuguese ICU. Port J Nephrol Hypertens.

[12] Samtleben W, Mistry-Burchardi N, Hartmann B, Lennertz A, Bosch T (2001). Therapeutic plasma exchange in the intensive care setting. Ther Apher.

[13] Melnick S, Nazir S, Gish D, Aryal MR (2016). Hypertriglyceridemic pancreatitis associated with confounding laboratory abnormalities. J Community Hosp Intern Med Perspect.

[14] Singh A, Shrestha M, Anand C (2016). Acute pancreatitis with normal amylase and lipase--an ED dilemma. Am J Emerg Med.

[15] Carvalho TJ, Martins AR, Calça R (2018). Therapeutic plasma exchange in the treatment of severe hypertriglyceridemia: a case report. Port J Nephrol Hypertens.

[16] Donelli D, Morini L, Trenti C, Santi R, Arioli D, Negri EA (2018). Plasma exchange for the treatment of transient extreme hypertriglyceridemia associated with diabetic ketoacidosis and acute pancreatitis. Eur J Case Rep Intern Med.

[17] Fei F, Boshell N, Williams LA 3rd (2020). Predictability and efficacy of therapeutic plasma exchange for hypertriglyceridemia induced acute pancreatitis. Transfus Apher Sci.

[18] Dehal H, Adashek M (2018). Total plasma exchange in hypertriglyceridemia-induced pancreatitis: case report and literature review. Case Rep Med.

[19] Cunha C, Barbosa AL, Pereira S, Barbosa L, Valente J, Fernandes J (2016). Plasma exchange in hypertriglyceridaemic acute pancreatitis: case report. Port J Nephrol Hypert.

[20] Song X, Shi D, Cui Q (2019). Intensive insulin therapy versus plasmapheresis in the management of hypertriglyceridemia-induced acute pancreatitis (Bi-TPAI trial): study protocol for a randomized controlled trial. Trials.

[21] Gubensek J, Andonova M, Jerman A (2022). Comparable triglyceride reduction with plasma exchange and insulin in acute pancreatitis - a randomized trial. Front Med (Lausanne).

[22] Mach F, Baigent C, Catapano AL (2020). 2019 ESC/EAS Guidelines for the management of dyslipidaemias: lipid modification to reduce cardiovascular risk. Eur Heart J.

[23] Costantini N, Mameli A, Marongiu F (2016). Plasmapheresis for preventing complication of hypertriglyceridemia: a case report and review of literature. Am J Ther.

[24] Francisco AR, Gonçalves I, Veiga F, Pedro MM, Pinto FJ, Brito D (2016). Hypertriglyceridemia: is there a role for prophylactic apheresis? A case report. J Bras Nefrol.

